# Research on Impact Resistance of Aluminum Alloy New Rotating Thin-Walled Structures

**DOI:** 10.3390/biomimetics8080590

**Published:** 2023-12-05

**Authors:** Shu-Cai Xu, Nuo Chen, Hao-Yi Qin, Rui-Xiang Wang, Xin Yang, Jia-Feng Song

**Affiliations:** 1 State Key Laboratory of Automotive Safety and Energy, Tsinghua University, Beijing 100084, China; xushc@mail.tsinghua.edu.cn; 2 Tsinghua University Suzhou Automobile Research Institute, Suzhou 215134, China; 13832240299@163.com (N.C.);; 3 College of Mechanical and Electrical Engineering, Hebei Agricultural University, Baoding 071001, China

**Keywords:** energy absorbing structure, gradient optimisation, impact resistance, rotating rigid structure, thin-walled

## Abstract

Honeycomb structures are widely used in the field of impact resistance and are constantly being developed and updated. In this paper, the design of three new aluminum alloy rotating thin-walled structures (NRTS) are examined. These structures combine common concave structures and rotating, rigid-body structures. The purpose of this study is to solve the problem of the poor energy absorption capacity of rotating, rigid-body structure due to small deformation and to provide a reference for honeycomb mechanism designs. The Young’s modulus, the critical velocity, and the platform stress of the NRTS structure are derived from theoretical analysis. The dynamic response of the NRTS structure at different impact velocities is investigated using finite element simulation software. The results show that the rotating, thin-walled recessed honeycomb (RTRH) increases the plateau stress by 124% and 51% as compared to rotating, thin-walled square tubes (RTSTs) and the re-entrant hexagonal structure (RH), respectively; the rotating, thin-walled quadruple-arc honeycomb structure (RTQH) increases the SEA by 21% and 20% as compared to the RTST and RH, respectively; and the rotating thin-walled double-arc honeycomb structure (RTDH) increases the CEF by 54% and 51% as compared to the RTST and RH, respectively. During the study, it was demonstrated that NTRS also exhibits good energy absorption capacity. Then, the effect of rotation angle on the energy absorption performance was analyzed. The cell and wall thickness of the NTRS structure were optimized according to the gradient theory. It was proved that the gradient optimized structure has better energy absorption performance as compared to the uniform structure.

## 1. Introduction

In the continuous development of structural design, strong quantitative and efficient energy-absorbing structures have always been highly sought after by engineers. Honeycomb structures are essential for designers to achieve this goal. Honeycomb structures are lighter in weight and have a higher specific strength than other structures. Porous structures have a larger porosity, providing more room for manoeuvre, which allows structural designers to create more structures with superior performances through the targeted design of porous structures. In recent years, a significant number of scholars have conducted a lot of research on honeycomb structures, resulting in high compression [1], highly efficient energy absorption [2], ablation resistance [3], acoustic performance [4], and many other excellent properties. Honeycomb structures are used in a wide range of applications in automotive, aerospace, biomedical, packaging, construction, and other fields.

Negative Poisson’s ratio structure, a special kind of lightweight honeycomb structure, exhibits superior performance [5]. Examples of its performance benefits include fracture resistance [6,7], penetration resistance [8], shear resistance [9,10], vibration and acoustic isolation properties [11,12], surface co-orientation [13], permeability variability [14], energy absorption properties, and impact resistance [15,16]. These characteristics makes these structures highly promising for a wide range of applications in aerospace, marine, mechanical automation, biomedical, defense and military, and textile industries.

Negative Poisson’s ratio structure is a typical structure composed of cellular units. The design of negative Poisson’s ratio structure is largely influenced by its cell design. Different cells have varying stiffnesses, deformation modes, and other characteristics. This design mode is more common in the design process of negative Poisson’s ratio structure. Many scholars have addressed the design of negative Poisson’s ratio structures, which are broadly categorized into concave structures, rotating rigid structures, chiral/anti-chiral structures, dot matrix structures, origami structures, etc. [17,18]. The structure design discussed in this paper is a rotating, rigid-body structure. A rotating, rigid-body structure consists of rigid units connected by flexible hinges, which produces a negative Poisson’s ratio effect through local rotation [17].

Energy absorption and impact resistance under quasi-static and dynamic loading are the primary concern for negative Poisson’s ratio structures. For the concave structure, Yu et al. [19] investigated the compression properties and damage behaviour of concave hexagonal honeycomb structures under composites, in which three types of composite expanding and contracting honeycomb structures were prepared using carbon/epoxy resin in order to compare the effect of gradient form on the mechanical properties. Compression experiments, finite element simulations, and theoretical analyses were conducted, resulting in the conclusion that the average expanding and contracting honeycomb has a better specific energy absorption compared with other gradient structures. Wang et al. [20] proposed a concave I-beam honeycomb structure with negative Poisson’s ratio effect in order to study its kinetic response characteristics, such as the deformation pattern and energy absorption of the concave I-beam honeycomb under different impact velocities. They compared the ortho-hexagonal honeycomb with the concave hexagonal honeycomb. The results showed that the concave I-beam honeycomb structure has a relatively lower peak stress and a longer plateau stress stage, and exhibited a better impact load consistency as well as better energy absorption characteristics with the increase of the impact velocity. Luo et al. [21] designed an antisymmetric curved cellular element based on the conventional concave hexagonal honeycomb cellular element, which is more shock-resistant than the conventional negative Poisson’s ratio honeycomb structure.

For rotational rigid structures, Grima and Evans [22] developed a rotationally designed structure in 2000. They demonstrated that modelled geometry is typically used as a planar projection in inorganic crystalline materials designed with octahedral coordination atoms. They also verified that the proposed new structure exhibited a negative Poisson’s ratio effect. Kusum et al. [23] proposed a new structure, analyzed using finite element modeling with experiments, and demonstrated that the new structure had a reduced stress concentration compared to recessed structures with similar dimensions and other geometric features. In order to enhance the mechanical properties of the negative Poisson’s ratio structure, Wang et al. [24] proposed rotating the conventional concave hexagon by a certain angle, based on the deformation mechanism of the rotating, rigid-body structure and the concave polygonal structure, to obtain a rotating concave hexagonal negative Poisson’s ratio structure. The dynamic properties of the rotating concave hexagon and the conventional concave hexagon were comparatively analyzed at different impact velocities. The results showed that the structural platform has a longer stress section and better energy absorption performance.

For the chiral structure, Wei et al. [25] obtained an inner-concave-anti-chiral honeycomb structure by combining an inner-concave hexagonal honeycomb with an anti-chiral honeycomb. They investigated the deformation patterns, critical impact velocities, platform strains, and platform stresses of the concave-anti-chiral honeycomb under different impact velocities using finite element software. The results showed that the concave-anti-chiral honeycomb exhibits a more pronounced negative Poisson’s ratio effect than the trilateral anti-chiral honeycomb. Li et al. [26] proposed a new auxiliary metamaterial: the folded star-shaped anti-chiral auxiliary metamaterial. They investigated the in-plane mechanical properties of the structure through experimental tests and numerical analysis, which showed the highest average compressive stress for the same thickness and same mass compared to the anti-tetradentate and the folded-star structure. For the point structure, Shen et al. [27] used the independent continuous mapping method for topology optimization to obtain a lightweight initial configuration. They designed a negative Poisson’s ratio energy-absorbing point structure in combination with a star-shaped structure, which was analyzed by using finite element simulation software; compared with the star-shaped point structure, the newly designed point structure has a higher energy-absorbing plateau load and larger plateau intervals. Sun et al. [28] designed a three-dimensional lattice structure to achieve the negative Poisson’s ratio effect based on the double-arrow negative Poisson’s ratio structure. The mechanical properties of the structure were comparatively analyzed for different parameters, including out-of-plane pressure, three-point bending, and low-velocity impact. Compared with the existing structures, the investigated structure has a smaller mass and superior impact absorption performance.

At present, most of the parent materials for 3D-printed negative Poisson’s ratio structures are metallic or non-metallic materials. Non-metallic materials such as ceramics and nylon have the advantages of light weight, cost-effectiveness, low sintering temperature, and easy processing and modification [29], and have been chosen by many scholars as the host materials for thin-walled junctions [5,30,31,32]. The advantages of mechanical strength, thermal and electrical conductivity, durability, and processability of metallic materials cannot be ignored [33,34], and likewise many scholars have chosen them as the parent materials for 3D printing [35,36]. In this study, the AA6061-T6 aluminum alloy, which is the most commonly used metal material, was selected as the base material [37], and a series of NTRS structures and traditional negative Poisson’s ratio structures (concave hexagonal structure and rotating, rigid-body structure) were prepared by Stereo Lithography Appearance (SLA) 3D printing technology.

In this study, to address the problem of the poor energy absorption capability in the rotating rigid body due to limited deformation [17], the rotating, rigid-body structure was combined with the common concave structure [19,38,39], and the aluminium alloy AA6061-T6 was used as the 3D printing material to design and manufacture a new type of rotating, thin-walled structure. Impact experiments with quasi-static and dynamic loading were performed on the structure along the in-plane. The reliability and scientific validity of the established finite element model were verified by comparing the data from numerical simulation and experiments. The energy absorption characteristics of the new rotating, thin-walled structure were also analyzed by comparing it with the energy absorption performance of the traditional structure.

## 2. Materials and Methods

### 2.1. Theoretical Analysis

This section presents a methodology for designing NRTS structures, describing the geometrical parameters of the structure and deriving the Young’s modulus of the structure and the relative density of the individual honeycomb structures. Additionally, the calculation of the critical velocity is given, and the prediction of the deformation modes at different velocities is provided.

#### 2.1.1. Young’s Modulus

**Figure 1:**
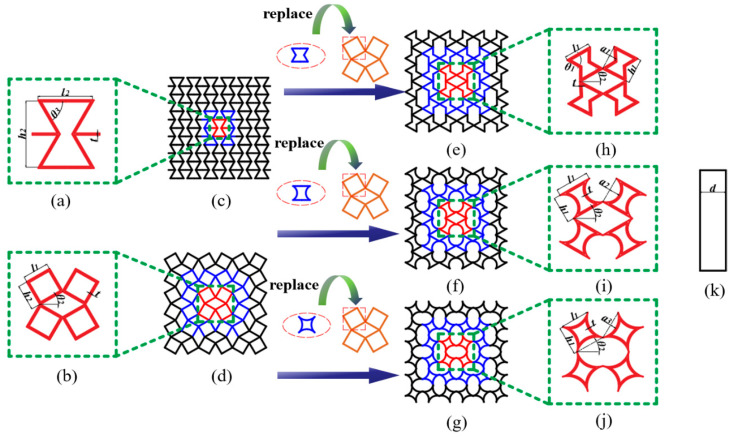
NRTS structural unit design and basic parameters. (**a**) RH represents the cell schematic and geometry; (**b**) RTST represents the cell schematic and geometry; (**c**) RH; (**d**) RTST; (**e**) RTRH; (**f**) RTDH; (**g**) RTQH; (**h**) RTRH represents the cell schematic and geometry; (**i**) RTDH represents the cell schematic and geometry; (**j**) RTQH represents the cell schematic and geometry; and (**k**) Out-of-plane thickness of honeycomb structure.

**Figure 2:**
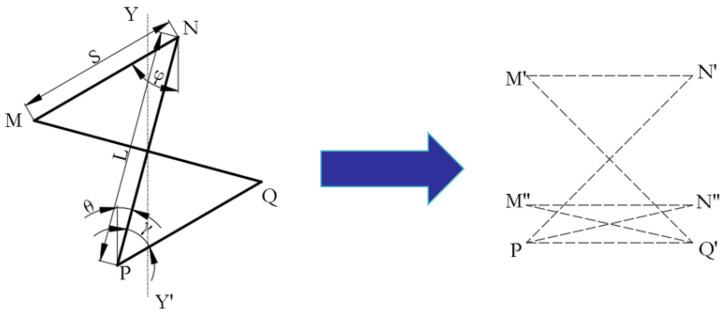
Simplified concave honeycomb structure with and without deformation.

The design concept of the new rotating, thin-walled structure is a honeycomb structure that is a combination of a traditional rotating, rigid-body structure and an inner-concave honeycomb structure, as shown in Figure 1. In order to facilitate the theoretical analysis, the three structures are simplified, as shown in Figure 2, and the inner concave hexagonal honeycomb is simplified into two triangles with deformations M′N′PQ′, as shown in Figure 2.

In the simplified cell, *γ* is the angle between PQ and PN, which acts as a spring hinge with a spring factor of *Kγ*, i.e., it is assumed that the cell changes only due to a change in the angle *γ* when loaded in the direction of YY′. the angle *φ* is the obtuse angle subtended by MN and the *Y*-axis, and the angle *θ* is the angle subtended by PN and the *Y*-axis. The rate of change of angle *φ* (Δ*φ*) is expressed as Δφ=kΔθ, where *k* is the ratio of the rate of change of angle *φ* (Δ*φ*) to the rate of change of angle *θ* (Δ*θ*):

(1) Δφ=kΔθ,

(2) dφ=kdθ,

According to [22], the following assumptions are madeas follows: the lengths of MQ and NP do not change at the beginning of compression, and PQ always moves only around the P point during deformation.

From the angle *φ* and angle *θ*, the cell size in the *XY* direction is:

(3) x=2Ssinφ−Lsinθ,

(4) y=Lcosθ,

The Poisson function is defined as follows:

(5) $$\nu_{\mathrm{xy}}=-\frac{d\varepsilon_{y}}{d\varepsilon_{x}},$$

where *dε_y_* and *dε_x_* in Equation (5) are the very small increments in the longitudinal and transverse directions, respectively, which are expressed as:

(6) $$d\varepsilon_{x}=\frac{dx}{x}=\frac{1}{x}\frac{dx}{d\theta }d\theta ,$$

(7) $$d\varepsilon_{y}=\frac{dy}{y}=\frac{1}{y}\frac{dy}{d\theta }d\theta ,$$

Equations (6) and (7) are brought into Equation (5) to obtain Equation (8)

(8) νxy=−dεydεx=−xy[dydθdxdθ],

where

(9) $$\frac{dy}{d\theta }=-L\sin\theta ,$$

(10) $$\frac{dx}{d\theta }=2S\cos\varphi \frac{d\varphi }{d\theta }-L\cos\theta ,$$

Bringing Equations (2), (9) and (10) into Equation (8) yields Equation (11)

(11) νxy=−dεydεx=−xy[dydθdxdθ]=tanθ(2Ssinφ−Lsinθ2Skcosφ−Lcosθ),

The Young’s modulus *E* of the designed structure can be derived using the energy conservation method and the strain energy due to incremental small strains *dε_x_* and *dε_y_* in the *XY* direction, as given by the following equation:

(12) $$U=\frac{1}{2}E_{x}{\left(d\varepsilon_{x}\right)}^{2},$$

(13) $$U=\frac{1}{2}E_{y}{\left(d\varepsilon_{y}\right)}^{2},$$

The work done by the rotating hinge on each cell is as follows:

(14) $$W=N[\frac{1}{2}k\gamma {\left(d\gamma \right)}^{2}],$$

where *N* is the number of hinges corresponding to a cell. Because each cell contains four cells, each cell contains four vertices, and two vertices correspond to one hinge: in this case, *N* = 8. Regarding the law of conservation of energy, Equations (12)–(14) are associated with the following equations:

(15) $$U=\frac{1}{V}W,$$

where *V* is the volume of the cell. Assuming a unit thickness of 1 in the third dimension, this volume is given by the following equation:

(16) V=xy,

Equation (17) is obtained by bringing Equations (6), (12) and (14) into Equation (15)

(17) Ex=8xkγy(dxdθ)2=8kγ(2Ssinφ−Lsinθ)Lcosθ(2Skcosφ−Lcosθ)2,

Equations (7), (13) and (14) are brought into Equation (15) to get Equation (18),

(18) Ey=8ykγx(dydθ)2=8kγcotθcscθL(2Ssinφ−Lsinθ),

where *E_x_* and *E_y_* are the Young’s modulus in the *X* and *Y* directions, respectively.

#### 2.1.2. Critical Speed

Under dynamic impact loading, the impact velocity is an important indicator that affects the dynamic response of cellular structures. When the impact velocity exceeds the trap velocity (first critical velocity) of the honeycomb structure, the structure starts to deform locally. The trapped velocity can be obtained from the following equation [40]:

(19) $$V_{cr1}=\int_{0}^{\varepsilon_{y}}c(\varepsilon )d\varepsilon ,$$

where *ε_y_* is the strain corresponding to reaching the peak of the first stress *c*(*ε*). It can be expressed as:

(20) $$c(\varepsilon )=\sqrt{\frac{\sigma^{\prime }(\varepsilon )}{\bar{\rho }\rho_{s}}},$$

where $\sigma^{\prime }(\varepsilon )$ is the modulus of elasticity of the honeycomb structure in its elastic phase, i.e., Young’s modulus, which has been derived in the previous section; *ρ_s_* is the density of the honeycomb matrix material; and $\bar{\rho }$ is the relative density of the honeycomb structure, which is demonstrated in Table 1 for different honeycombs.

With the gradual increase of the velocity, the deformation mode of the honeycomb structure changes from local deformation to layer-by-layer collapse, and its corresponding critical velocity is called the second critical velocity, which is expressed as follows [41]:

(21) $$V_{cr2}=\sqrt{\frac{2\sigma_{0}\varepsilon_{0}}{\bar{\rho }\rho_{s}}},$$

where *σ*_0_ is the static yield stress of the honeycomb structure and *ε*_0_ is the densification strain; theoretically, the densification strain will simply be equal to the porosity, but in the experiments, it has been found that the densification strain is less than the porosity [42]. The densification strain can be calculated by an expression with empirical coefficients:

(22) $$\varepsilon_{0}=1-\lambda \bar{\rho },$$

where *λ* is a factor that depends on the honeycomb structure in the range λ∈[1,3].

Based on the critical velocity, we can speculate the deformation mode:

At time $v<V_{cr1}$, the honeycomb structure is in the overall deformation mode.

At time $V_{cr1}<v<V_{cr2}$, the honeycomb structure is in local deformation mode.

And at time $V_{cr2}<v$, the honeycomb is in layer-by-layer collapse deformation mode.

### 2.2. Experiments

In this section, samples were fabricated using 3D printing and the basic parameters of each honeycomb sample were counted. Quasi-static compression experiments were performed on the printed specimens and data were obtained for subsequent verification that the finite element model is valid.

#### Quasi-Static Compression Experiment

**Figure 3:**
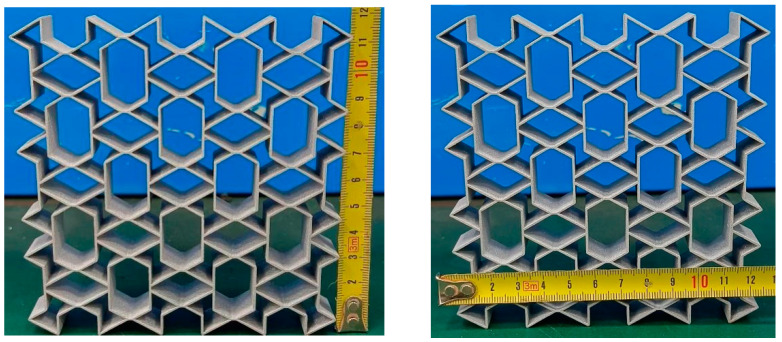
Samples of the RTRH structures fabricated using 3D printing.

Currently, the substrates used in 3D printing are mainly divided into three categories: metal materials, organic polymer materials, and inorganic non-metallic materials [43]. Among them, metal 3D printing technology can achieve the advantages of functional integration, lightweightness, topology optimisation, and so on [44]. A large number of scholars have studied the mechanical properties of materials or structures through compression experiments [45,46,47,48,49,50,51]. In this study, the AA6061-T6 aluminium alloy [37], which is the most commonly used metal material, was selected as the base material, and 3D printing was used to prepare the specimens and test their mechanical properties through quasi-static compression experiments. Properties of the AA6061-T6 aluminium alloy material include a Young’s modulus of 68.2 GPa, a material density of 2.7 × 10^3^ kg/m^3^, a Poisson’s ratio of 0.33, and a yield stress of 251.52 MPa [37]. Three-dimensionals models of all specimens were constructed in SolidWorks and imported into stereo lithography appearance (SLA) 3D Printers in an STL file format. The samples were fabricated using the SLA technique with the thickness of the printed layer set at 0.1 mm and a support structure. Metal powder printing was used in this study. It was solid after printing, so there was no infill. The printing direction was perpendicular to the sample compression direction (perpendicular to the surface direction shown in Figure 3).

The RTRH structure with 3 × 3 unit elements in the *x* × *z* plane is shown in Figure 3. The RTRH structure had the same cell dimensions: each cell was 40 mm in length, 40 mm in height, and 20 mm in width. The cells had the same overall dimensions as the RH structure, and the specific dimensions of each sample are shown in Table 2. The complete dimensions of the specimens were 120 × 120 × 20 mm.

**Figure 4:**
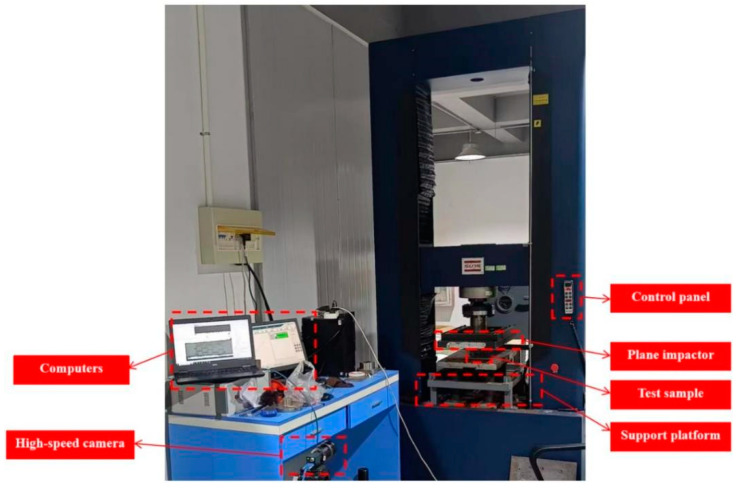
Universal Testing Machine.

The quasi-static compression test was carried out using a Universal Testing Machine (UTM5504X-WGDN, Shenzhen, China), which consists of measuring systems, drive systems, control systems and computers, etc., as shown in Figure 4. The specimen was compressed at a rate of 5 mm/min.

### 2.3. Numerical Modelling

This section describes the process of finite element modelling and reliability analysis, including dimensions, materials, boundary conditions, and cell types of the honeycomb structure. The mesh dimensions were determined by mesh convergence analysis and the finite element simulation results were compared with the test results to ensure the validity of the work.

#### 2.3.1. Establishment of Finite Element Modelling

**Figure 5:**
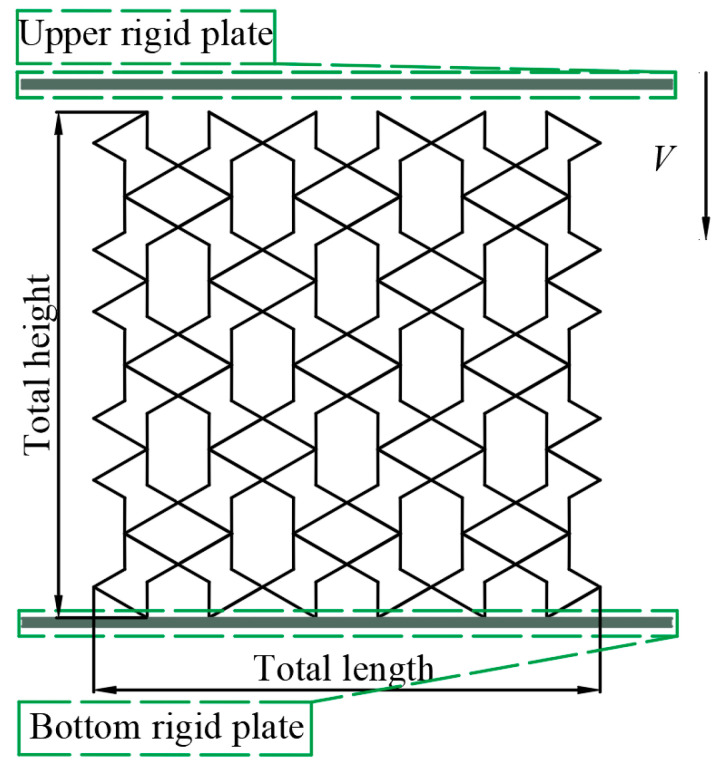
Schematic diagram of the computational model of the RTRH structure under axial impacts.

The in-plane compression simulation of the honeycomb structure was carried out using the finite element simulation soft. The pre-processing chosen was Hypermesh 2019, and the solver used LS-DYNA software, specifically version R11.0.0 Parallel. A schematic of the computational model of the RTRH structure under longitudinal impact is shown in Figure 5. The upper and lower plates are rigid plates with a density of 7.8 × 103 kg/m^3^ and a Young’s modulus of 210 GPa, and the material of the plates is steel. The upper plate was given a constant velocity *V* = 1 mm/ms along the negative direction of the *Y*-axis, and the upper plate as well as the RTRH structure, the RTRH structure, and the lower plate were in automatic face-to-face contact, with a kinetic friction coefficient of 0.2 and a static friction coefficient of 0.3. In practical conditions, the deformation of the indenter and the support is very small and is usually neglected;therefore, a 20-gauge material card setup was used with the stiffener properties.

The structure had a total length of 120 mm, a total height of 120 mm, and a total width of 20 mm. The calculation was performed by using the Belytschko-Tsay shell unit formula; the number of nodes was four, the number of points of integration was two, and it was set up by using the No. 24 (linear elasticity) material card. The base material is AA6061-T6 aluminium alloy with a density of 2.7 × 103 kg/m^3^, a Young’s modulus of 68.2 GPa, a Poisson’s ratio of 0.33, and a yield strength of 251.52 MPa [37].

**Figure 6:**
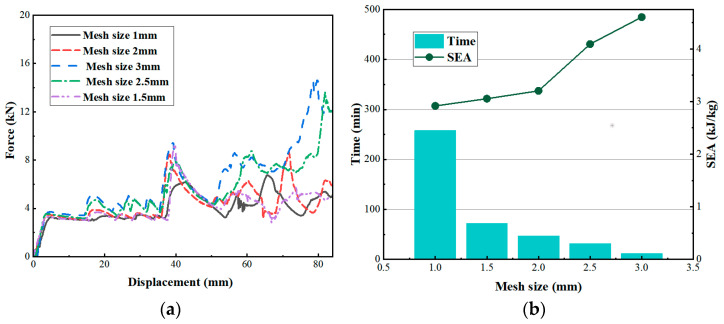
Grid sensitivity analysis. (**a**) Force-displacement curves with different mesh sizes and (**b**) Variation of calculation time and specific absorption energy for the different grid sizes.

The sensitivity analysis of the model was conducted under the premise of ensuring the accuracy of the calculation and minimizing the computational cost [37]. Mesh with side lengths of 1 mm, 1.5 mm, 2 mm, 2.5 mm and 3 mm were chosen to divide the finite element model, respectively. Figure 6a shows the force-displacement curves for different mesh sizes, and it can be seen that the force-displacement curves show a converging trend when the mesh size is 2 mm or smaller. This is corroborated by the variation of the specific absorption energy SEA at different grid sizes in Figure 6b. The variation of the specific absorption energy decreases when the mesh size is 2 mm or smaller. From Figure 6b, it can be seen that the computational time increases significantly when the mesh size is within 1.5 mm. Combining the convergence speed and the time required for computation, the mesh size of 1.5 × 1.5 mm was chosen to divide the finite element model in this paper. After the grid was divided, there were 23,193 elements of RTRH.

#### 2.3.2. Validation of the Finite Element Model

**Figure 7:**
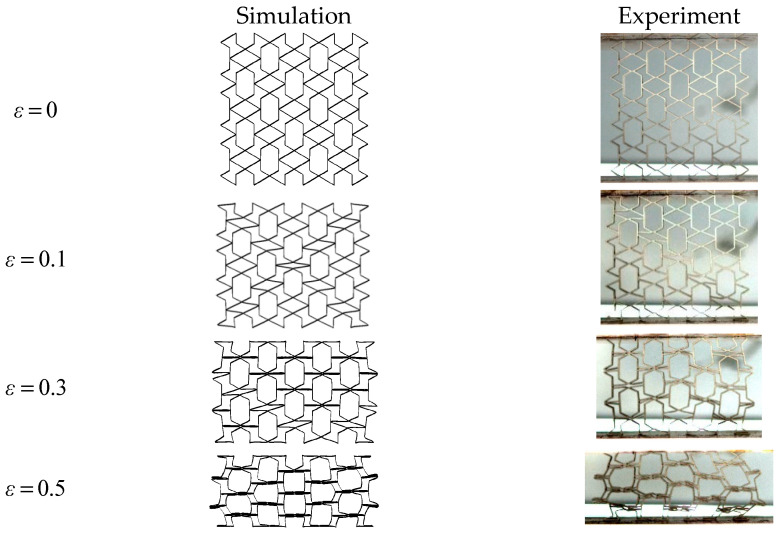
Comparison between the experimental specimen and the simulated deformation.

**Figure 8:**
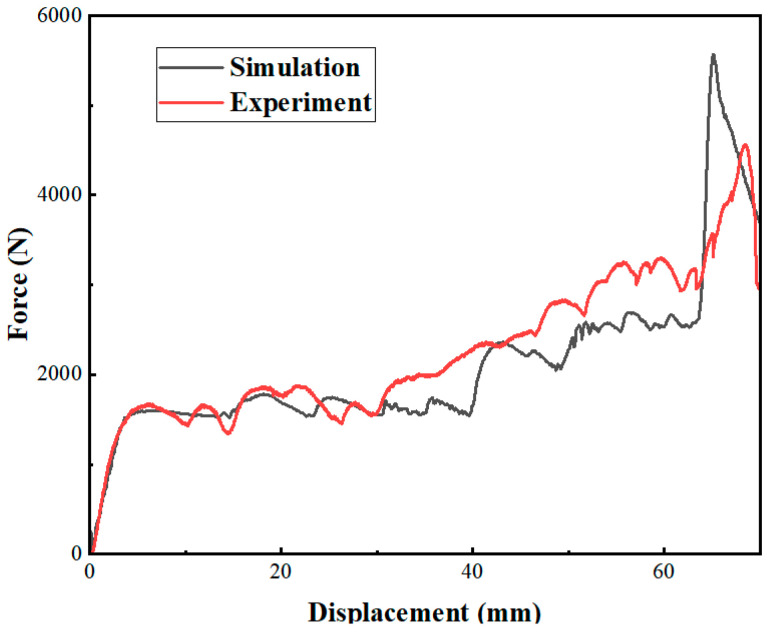
Stress-strain curve of the experimental specimen and the simulation.

Figure 7 demonstrates the deformation pattern of the honeycomb structure under quasi-static compression. Through the comparison of the simulation and the experiment, it is not difficult to find that the deformation of the specimen in the finite element model simulation and the compression experiment are the same. This proves the rationality of the experiment and the validity of the simulation. Figure 8 shows the response curves of the different honeycomb structures in the finite element simulation and compression experiments; from the overall trend, the experimental and simulation results are basically the same, and the maximum error is 6.6%, which is within the acceptable range. Therefore, the finite element model established in this paper can effectively simulate real working conditions. The studies that follow in this paper are based on the conclusions drawn after finite element simulation.

## 3. Results and Discussion

In this section, the energy absorption properties and deformation modes of the NRTS structure at different velocities were analyzed along with the dynamic response of the rotation angle to the NRTS. The gradient optimisation design of the NRTS structure cell and wall thickness was also conducted by combining the gradient theory.

### 3.1. Energy Absorption Properties of Honeycomb Structures

**Figure 9:**
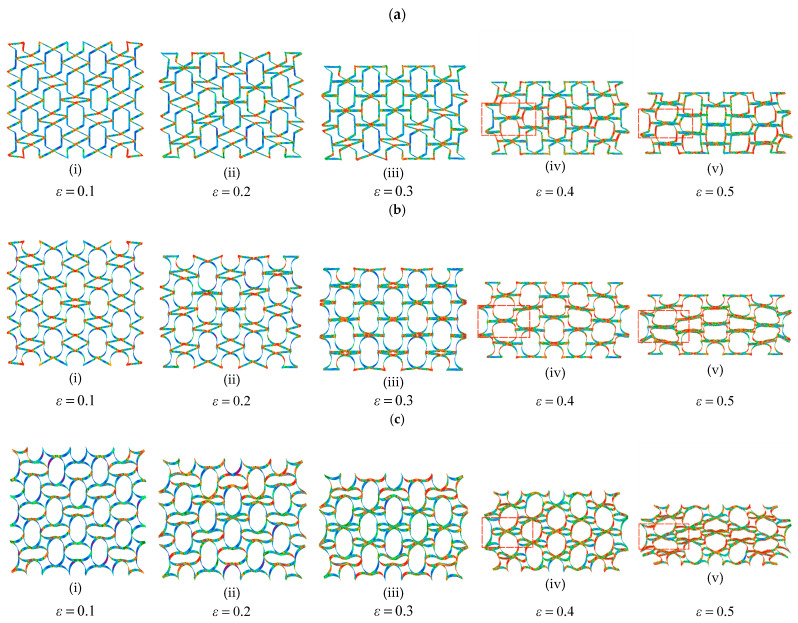
Deformation of the NRTS structure. (**a**) Deformation and stress cloud of the RTRH structure, (**b**) deformation and stress cloud of the RTDH structure, and (**c**) deformation and stress map of the RTQH structure.

Figure 9 shows the deformation process of the NRTS structures under different strains. As shown in Figure 9, the RTRH, RTDH, and RTQH structures underwent rotational deformation first, and the pores that formed between the cells gradually decreased until they disappeared. Then, they were compressed in the longitudinal direction, which is the same as the deformation pattern predicted in Section 2.1. As can be seen in Figure 9, the rotational deformation process was not simultaneously rotational or layer-by-layer, but the diagonal cells were firstly rotationally deformed. Its deformation pattern is similar to the “X”-shaped deformation zone of the concave hexagon [15]. There is no obvious sign of expansion during the rotational deformation process, which is consistent with the deformation mode of the negative Poisson’s ratio structure of a rotating rigid body [17]. As the longitudinal compression continued, the larger pores in compression showed a tendency to expand, the honeycomb structure expanded outward, and the negative Poisson’s ratio effect of the RTRH, RTDH, and RTQH structures disappeared; the characteristics of the rotationally deformed structures will be described later. As shown in the red square area in Figure 9, the deformation of the inner side of the cell was much larger than that of the outer side, and there was a larger stress; it is easy to see that the compression deformation stage started from the inner side first.

For elasto-plastic honeycomb structural bodies such as metals and many polymers, plastic collapse occurs when the hole wall bends to the full plastic moment and a plateau occurs at the plastic collapse stress, which is then called the plateau stress. For honeycomb structures, the platform stress is an important indicator of the impact, and the mechanical properties of the honeycomb can be expressed by the following equation [52]:

(23) $$\sigma_{p}=\frac{1}{\varepsilon_{d}-\varepsilon_{y}}\int_{\varepsilon_{y}}^{\varepsilon_{d}}\sigma (\varepsilon )d\varepsilon ,$$

where $\varepsilon_{d}$ is the structural compaction strain.

**Figure 10:**
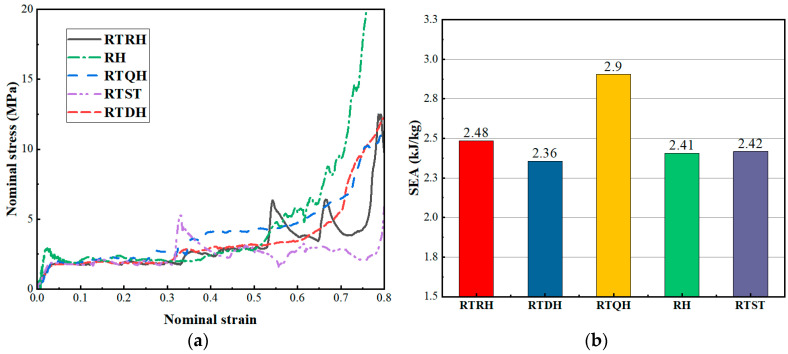
Energy absorption properties of each honeycomb structure. (**a**) Stress-strain curve of each honeycomb structure, and (**b**) specific absorption energy of each honeycomb.

The platform stress of each honeycomb structure can be calculated from Figure 10a, and the calculation results are shown in Table 3. Because there are two platform stresses in the rotating rigid body structure, the total platform stress of its structure is the sum of the two platform stresses. From Table 3, it can be seen that the platform stress of the rotating rigid body structure was larger than that of the RH structure, in which the RTQH structure and the RTRH structure had larger platform stresses that increased by 78% and 124%, respectively, as compared to the RH structure, and by 20% and 51%, respectively, as compared to the RTST structure.

Specific energy absorption (SEA), peak force (PF), and average crushing force Efficiency (CFE) are also commonly used to evaluate the impact design of honeycomb structures [53].

The SEA refers to the capacity of a structure to absorb energy per unit mass and is given by the following formula:

(24) $$SEA=\frac{EA}{m}=\frac{\int_{\varepsilon_{y}}^{\varepsilon_{d}}\sigma (\varepsilon )d\varepsilon }{\bar{\rho }\rho_{s}},$$

where *m* is the mass of the honeycomb structure and *EA* is the energy absorbed by the structure during the crushing process. The latter can be calculated from the following equation:

(25) $$EA=\int_{a}^{b}f(x)dx,$$

where *f*(*x*) is a function of load as a function of displacement, and *a* and *b* are the starting and ending points for calculating the energy absorption value.

*CFE* is a measure of the stability of energy absorption of the structure during the compression collapse process and is calculated by the following formula:

(26) $$CEF=\frac{\bar{F}}{PF}\times 100\%,$$

(27) $$\bar{F}=\frac{EA}{b-a},$$

where represents the average load in the interval (*a*,*b*) and the maximum load in that interval.

**Figure 11:**
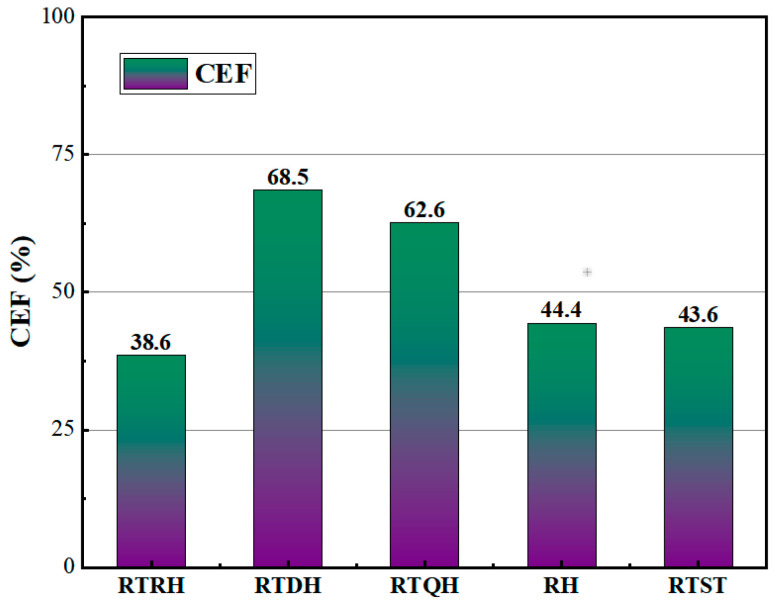
Average crush force of each honeycomb structure under quasi-static compression.

Based on the above expression, the trend of the SEA with strain for each honeycomb structure obtained in the quasi-static compression test is shown in Figure 10b, and Figure 11 illustrates the CEF of each honeycomb structure during the compression collapse process. From Figure 10b, it can be seen that the NRTS structures have good energy absorption capacity, and the RTQH structure improves the specific energy absorption by 21% and 20%, respectively, as compared to the conventional concave honeycomb structure and rotating rigid body structure.

This is due to the NRTS’s four-sided curved structure, which at the end of the rotational deformation process still has more pores as compared to the other structures and shown in Figure 9c; because of the presence of these pores, a larger force is required to compress the RTQH structure, allowing the structure to absorb more energy before densification strain.

From Figure 11, it can be seen that the RTDH structure has a good compressive collapse force efficiency, starting from a CEF enhancement of 54% and 57%, respectively, as compared to the conventional honeycomb structure and the rotating rigid body structure. The higher CEF of the RTQH structure is also due to the fact that, after the rotational deformation of the RTDH, the curved side edges in the cell form superior arcs with the neighbouring cells, which are more stable in compression than the the tetragonal shape.

### 3.2. Deformation Patterns of Honeycomb Structures at Different Velocities

The energy absorption properties of honeycomb structures are more sensitive to changes in velocity. At different impact velocities, honeycomb structures often exhibit different deformation patterns and energy absorption capabilities. In this paper, the expression for the critical velocity is derived in the theoretical analysis section. Based on the above study, three velocities of 1 m/s, 30 m/s and 100 m/s were chosen to represent the low-, medium- and high-impact velocities for the NRTS structure. The deformation pattern and energy absorption capacity of the honeycomb structure were analysed at the three impact velocities. Because the selected aluminium alloy material was shown to have no significant effect on the stresses at strain rates below 103 s^−1^ [54] and the maximum strain rate of the selected velocity in this paper was 833 s^−1^, the strain rate effect was not considered in the simulation analysis.

**Figure 12:**
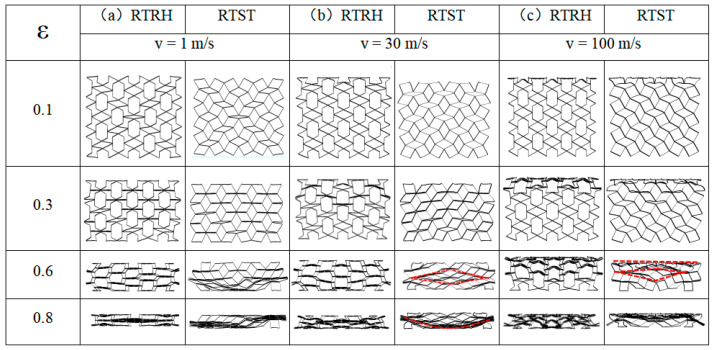
Deformation patterns of the RTRH and RTST at different impact velocities, ε is the nominal strain, which is defined as the ratio of the compression distance to the total length of the honeycomb structure. (**a**) Deformation patterns of RTRH and RTST at 1 m/s impact velocity, (**b**) deformation patterns of the RTRH and RTST at 30 m/s impact velocity, and (**c**) deformation patterns of the RTRH and RTST at 100 m/s.

Because many scholars have already conducted relevant studies on the deformation modes and energy absorption characteristics of RH structures under different impact velocities [15,55], this paper focuses on the dynamic loading of rotating rigid-body structures and does not describe the deformation modes of RH structures. As shown in Figure 12a, the RTRH structure first undergwent cell rotation deformation under the low-velocity loading process by rotating the diagonal cells first. This is similar to an “X”-shaped deformation band and is a common deformation for rotating rigid body structures. This deformation has already been described in the previous section, so it will not be repeated in this section. When the rotational deformation ended, the inner concave hexagonal cell was impacted and two types of tetragonal pores of different sizes were formed. Due to the instability of the tetragonal shape, the structure changed to folding deformation as the compression continued, i.e., the small tetragonal pores were gradually tilted until they coincided with the bottom edge. Finally, the RTRH structure entered the densification phase. At this point, the honeycomb structure was “destructively” crushed and the structure was no longer energy-absorbing. The RTST structures were first crushed at the end of the rotational deformation phase when the lower cells were first crushed, and then were crushed layer by layer, from the bottom to the top, until they finally entered the densification phase.

**Figure 13:**
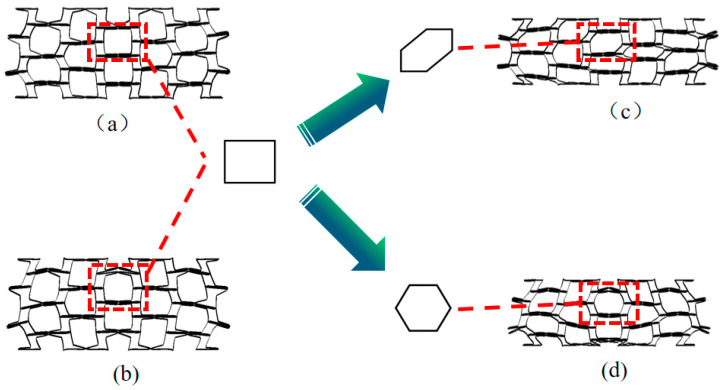
Differences in pore changes of the RTRH structure at different velocities. (**a**) Deformation of RTRH at 1 m/s velocity with ε = 0.5, (**b**) deformation of the RTRH at 30 m/s velocity with ε = 0.5, (**c**) deformation of the RTRH at 1 m/s velocity with ε = 0.6, and (**d**) deformation of the RTRH at 30 m/s velocity with ε = 0.6.

Figure 12b shows the deformation patterns of the RTRH structure and the RTST structure at medium-velocity impacts. At the beginning of the impact, the RTRH structure differed from the deformation pattern at low velocity. The honeycomb structure started to undergo compressive deformation before the rotational deformation was finished. In the sub-stage, the RTRH structure showed a tendency to expand in the longitudinal direction, and the negative Poisson’s ratio effect was weakened. As the compression continued and the rotational deformation ended, the RTRH structure changed from layer-by-layer parallel folding to inward folding under the influence of the increasing impact velocity. Figure 13 plots the deformation sketches of the pores at two different impact velocities. When the strain reached 0.8, the honeycomb structure entered the densification stage. In the early stage of strain, the RHST structure, like the RTRH structure, received compression of the cells while rotationally deforming. When the rotational deformation ended, the RTST structure presented a shuttle-shaped deformation zone, as shown in Figure 12b, and started to compress from the inner side. When the strain was 0.8, the RTST structure basically entered the densification stage, and the deformation pattern changed to a “U”-shaped deformation zone.

The deformation pattern of the honeycomb structure under high-speed impact had a mostly “I”-shaped deformation zone. Figure 12c shows the deformation patterns of the RTRH and RTST at high speeds, which were approximately the same. When the strain was small, the “I”-shaped deformation band was formed at the impact end of the two structures, and it expanded downward with the increase of strain. When the strain reached 0.8, the RTST structure formed a shuttle-shaped deformation zone under the “I”-shaped deformation zone until the honeycomb structure entered the dense strain stage. As the impact velocity of the honeycomb structure exceeded the second critical velocity, and the honeycomb structure collapsed layer by layer, the cell outside the deformation zone was less deformed. Therefore, the negative Poisson’s ratio effect of the rotating rigid body structure was basically lost.

The “X”-shaped deformation zone, the “U”-shaped deformation zone, and the “I”-shaped deformation zone described in this paper are the deformation patterns of honeycomb occurring at different velocities [56], which is a kind of people’s description of the deformation of honeycomb structures. The deformation patterns of different honeycomb structures also differed at low and medium speeds [25,57,58]. However, under the high-speed impact condition, the inertia effect of the honeycomb structure was enhanced, and the deformation zone was mainly concentrated at the impact end, which corresponds to the “I”-type, layer-by-layer collapse deformation pattern from the impact end to the stationary end [59], which is common to almost all honeycombs [25,57,58].

**Figure 14:**
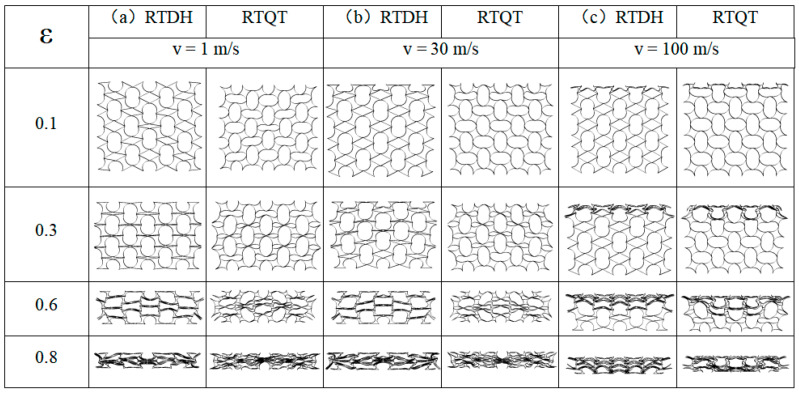
Deformation patterns of the RTDH and RTQT at different impact velocities: ε is the nominal strain, which is defined as the ratio of the compression distance to the total length of the honeycomb structure. (**a**) Deformation patterns of the RTDH and RTQT at 1 m/s impact velocity, (**b**) deformation patterns of the RTDH and RTQT at 30 m/s impact velocity, and (**c**) deformation patterns of the RTDH and RTQT at 100 m/s.

The deformation patterns of the RTDH and RTQH at different impact velocities are shown in Figure 14. Figure 14a shows the deformation patterns of the two honeycomb structures at low-impact velocities. The deformation pattern is the same as that of the rotating rigid body structure, which is divided into two stages, i.e., the rotational deformation stage and the longitudinal compression collapse stage. The rotational deformation stage has been described in the previous section, and this section mainly describes its deformation pattern during the longitudinal compression collapse.

The RTDH structure began to expand after the end of the rotational deformation, when its larger elliptical pores were compressed in the longitudinal direction. From the deformation image of ε = 0.6 in Figure 14a, it can be seen that the deformation is most obvious at the middle position of the honeycomb structure, and the deformation shapes are similar to that of the RTRH at ε = 0.6 at 30 m/s. The deformation shapes are all in the shape of positive hexagon, and the side edges of the elliptical pores are folded inward for compression. Until the strain was 0.8, the honeycomb structure entered the densification stage. The RTQH structure has more pores though, which gives it a good deformation capacity. However, the deformation was the same as that of the RTDH structure at a low-velocity impact. Both structures deformed from the layer side, and the deformation pattern was similar to a positive hexagon. This deformation pattern lasted until the loss of energy absorption capacity in the densification stage. The medium-velocity impact is shown in Figure 14b, where the increase in impact velocity exceeds the first critical velocity of the honeycomb structure. The RTDH and RTQH structures underwent compressive deformation before the rotational deformation was completed, from the previous overall deformation mode to the local deformation mode. This finding is consistent with the assumptions made in the theoretical stage of this paper. The RTDH structure did not change significantly during the longitudinal compression of the structure with low-velocity impact. However, in this stage of the deformation process, the deformation of the upper side of the RTDH structure was larger than that of its lower side at a strain of 0.8 as compared with that of the low-velocity impact. This is due to the impact velocity exceeding the first critical velocity. Meanwhile, this difference in deformation confirms that the honeycomb structure changed from the previous overall deformation to localised deformation under medium-velocity impact. Figure 14c. shows the deformation patterns of the two structures at high-impact speeds. The RTDH and RTQH structures were subject to significant inertial effects, and the honeycomb only exhibited an “I”-shaped deformation band from impact to complete densification.

**Figure 15:**
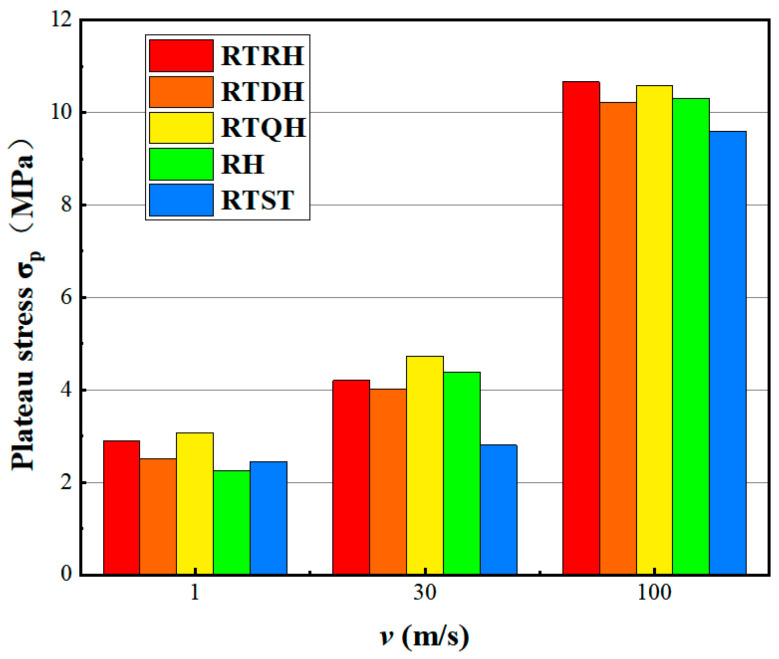
Comparison of platform stresses at different velocities for each honeycomb structure.

In order to compare the impact resistance of different honeycombs, Figure 15 shows the stress histograms of the RTRH, RTDH, RTQH, RH, and RTST structures at different impact velocities. It can be seen that the platform stress of the NTRS structure was significantly larger than that of the RTST structure. The RTQH structure had excellent plateau stress at low- and medium-impact speeds. At high speeds, the RTRH structure had a higher plateau stress as compared to the other structures. This indicates that the RTRH structure has a good energy absorption performance under high-speed impact. The platform stress of the different honeycombs increased with the increase of impact velocity.

### 3.3. Impact of Parametric Analysis and Gradient Optimisation

In the previous section, it was shown that the NRTS structure has good energy absorption capacity. Therefore, in this section, the NRTS structure was chosen as the base structure to investigate the effects of different angles and combinations of shapes on the energy absorption capacity of the structure.

#### 3.3.1. Effect of Different Rotation Angles

**Figure 16:**
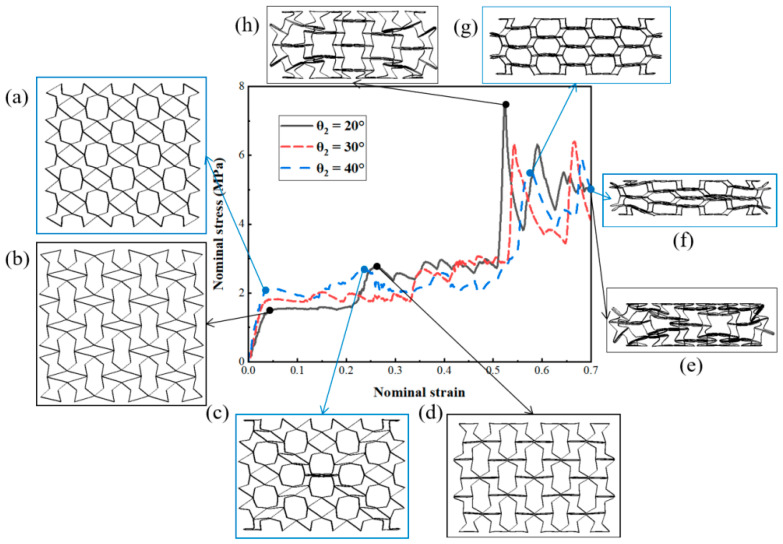
Stress-strain curves and deformation patterns of the RTRH structure at different rotation angles at an impact velocity of 1 m/s. The RTRH-20° is represented by the black curve and the RTRH-40° by the blue curve. The blue wireframe shows the deformation patterns of RTRH-40° at different strains: (**a**) ε = 0.04, (**c**) ε = 0.24, (**g**) ε = 0.58, and (**f**) ε = 0.7. The black wireframe shows the deformation patterns of the RTRH-20° at different strains: (**b**) ε = 0.06, (**d**) ε = 0.26, (**h**) ε = 0.52, and (**e**) ε = 0.7.

In this study, when designing structural units, it was found that different angles of *θ*_2_ had obvious effects on the structural deformation for the RTRH structures. When the rotation angle *θ*_2_ was less than 30°, the RTRH structure exhibited obvious signs of contraction after pore compression, indicating a significant negative Poisson’s ratio effect, but its rotational deformation phase was shortened; when *θ*_2_ is greater than 30°, the rotational deformation phase of the RTRH structure increased, but the structure showed a trend of expansion during compression, and the negative Poisson’s ratio phenomenon was reduced. Therefore, this section focuses on the effects of the different rotation angles of the RTRH structures on the energy absorption performance under dynamic impact. The rotation angles *θ*_2_ were designated as 20°, 30°, and 40°, and for the convenience of the study, the total length *l* = 120 mm, the total height *h* = 120 mm, the width *d* = 20 mm, and the wall thickness *t* = 1 mm for the three structures, as shown in Figure 16a,b.

**Figure 17:**
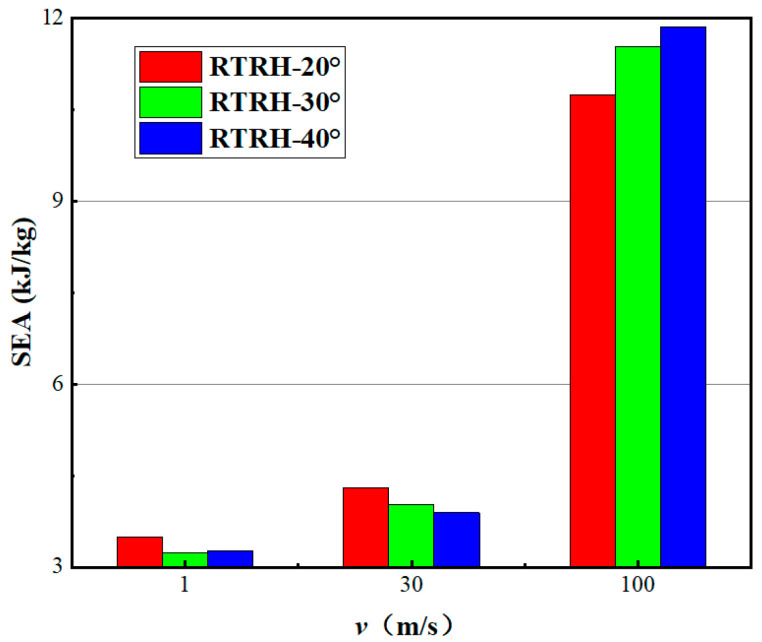
Comparison of platform stresses at different speeds for each rotation angle of the RTRH structure.

Figure 16 illustrates the stress-strain curves of the RTRH at rotation angles of 20° and 40°. Figure 16c illustrates the deformation of the RTRH-40° structure at the onset of the second plateau stress, and it can be seen that, due to the increase in the rotation angle, the compressive deformation of the crystal cell had already begun before the rotational deformation had ended. Please refer to Figure 16g: Deformation of RTRH-40° at ε = 0.57. The deformation modes were folding and compression layer by layer until it entered the densification strain, as shown in Figure 16f. The rotational deformation of the RTRH-20° structure was completed when ε = 0.26, as shown in Figure 16d. The RTRH-20° then started to undergo compressive deformation. Figure 16h shows its deformation during compression, and it can be seen that the structure had a tendency to expand. Due to the small rotation angle, the cellular connection was tight and its stress increase was rapid during the sub-process. Subsequently, the negative Poisson’s ratio effect of its RTRH-20° was revealed. It showed an inward contraction trend until it entered the densification strain stage and finally lost its energy absorption capacity. Figure 17 demonstrates the specific energy absorption of the RTRH structure for three rotation angles at different speeds. It can be seen that the specific energy absorption of RTRH-20° was larger than that of the other two structures at low-speed impacts, and the negative Poisson’s ratio effect of RTRH-20° can absorb more energy as compared with the deformation of the rotating rigid body. In the medium-speed impact, the absorbed energy of the RTRH-40° did not increase significantly as compared with the other two structures, and its specific absorbed energy was lower than that of the other two structures; at this time, the negative Poisson’s ratio effect of the RTRH-20° still played an obvious role, as shown in Figure 17b. However, in the case of high-speed impact, as shown in Figure 17c, the negative Poisson’s ratio effect of the RTRH-20° was lost due to the inertia effect caused by the large impact speed, and the strain curve of the specific absorption energy in Figure 17c shows that the specific absorption energy of RTRH-20° was always lower than that of the other structures, and the specific absorption energy of RTRH-40° had a good specific absorption energy in the case of high-speed impact.

Table 4 and Table 5 show the histograms of the specific energy absorption of the RTDH and RTQH structures at different rotation angles, respectively. It can be seen that the specific energy absorption of the RTDH and RTQH structures did not differ much in the low- and medium-speed impact stages because the deformation pattern did not significantly change when the rotation angle was changed. In the high-speed impact stage, the difference in the rotation angle started to appear, and the RTDH-40° structure and RTQH-30° structure showed good energy absorption performance.

#### 3.3.2. Cell Gradient Optimisation

**Figure 18:**
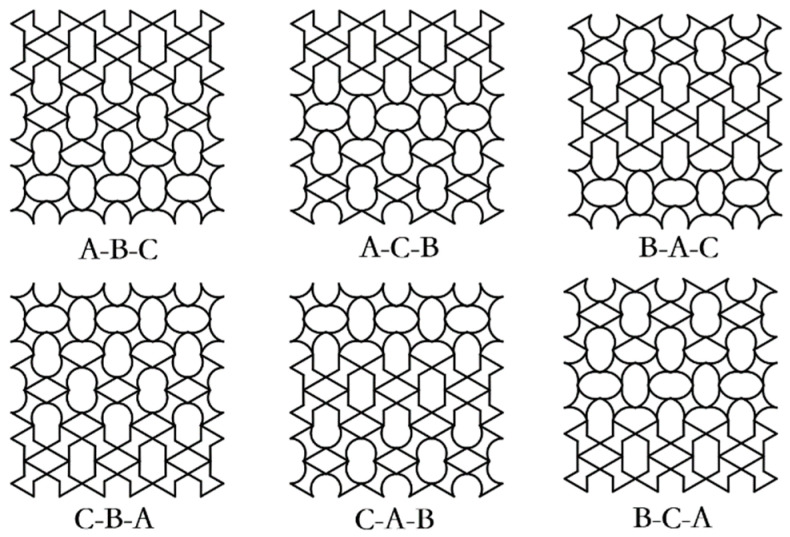
NRTS hybrid gradient mode.

**Figure 19:**
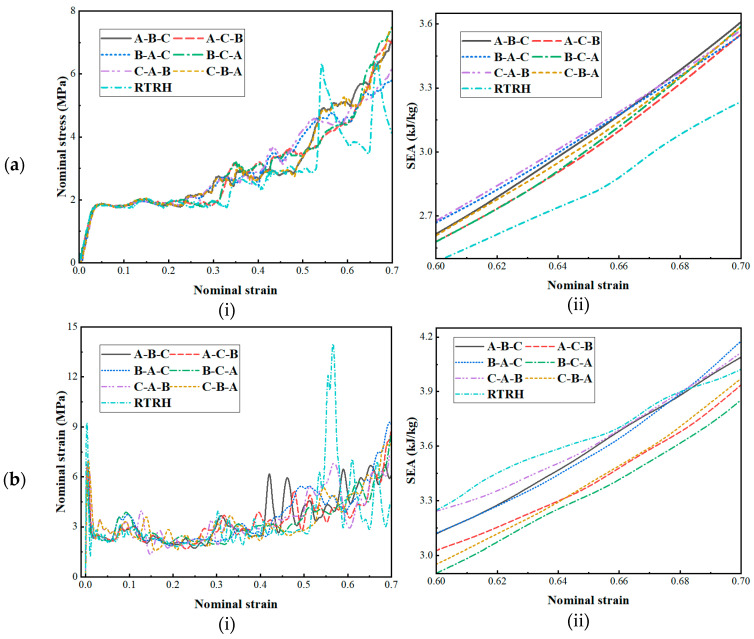

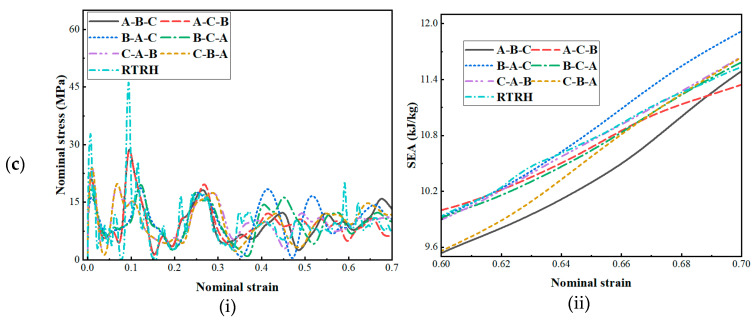
Dynamic response of the NRTS grain cell gradient: (**a**) *v* = 1 m/s, (**b**) *v* = 30 m/s, and (**c**) *v* = 100 m/s.

In this section, a gradient design was performed based on the underlying geometric cell of the NRTS structure, whose ensemble model is shown in Figure 18. For the convenience of the description of the article, the RTRH cell is denoted as A, the RTDH cell as B, and the RTQH cell as C. The designed hybrid gradient model was compared with the single cell structure under low-, medium-, and high-speed impact simulations, and the dynamic response was obtained, as shown in Figure 19.

Figure 19a–c shows the stress-strain curves and specific energy absorption strain curves of the gradient design structures and the RTRH structures for low-, medium-, and high-velocity impact simulations. From Figure 19a, it can be seen that at low speeds, the stress difference between the gradient-designed structures was not very large, but there was an obvious difference with the single-cell structure; from the specific energy-absorption strain curves, it can be seen that there was no big difference in the energy absorption of the six gradient-designed structures, which were all higher than that of the RTRH structure. However, during the medium-velocity impact, the differences between the structures began to appear, as shown in Figure 19b. Among them, the B-A-C structure, the A-B-C structure, and the B-A-C structure demonstrated better energy absorption performances than the single-cell structure. As the velocity increased further, as shown in Figure 19c, the difference between the structures further increased, and it can be seen from the strain curves of the specific energy absorption that the specific energy absorption of both the B-A-C structure and the C-B-A structure was slightly higher than that of the single-cell structure. Figure 19 demonstrates that the reasonably designed mixed-cell gradient model has better energy absorption performance than the single-cell model under dynamic impact.

#### 3.3.3. Thickness Gradient Optimisation

**Figure 20:**
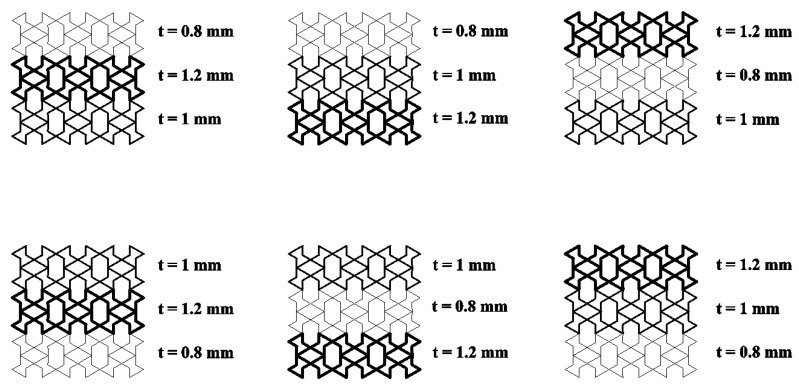
RTRH structural wall thickness gradient design.

**Figure 21:**
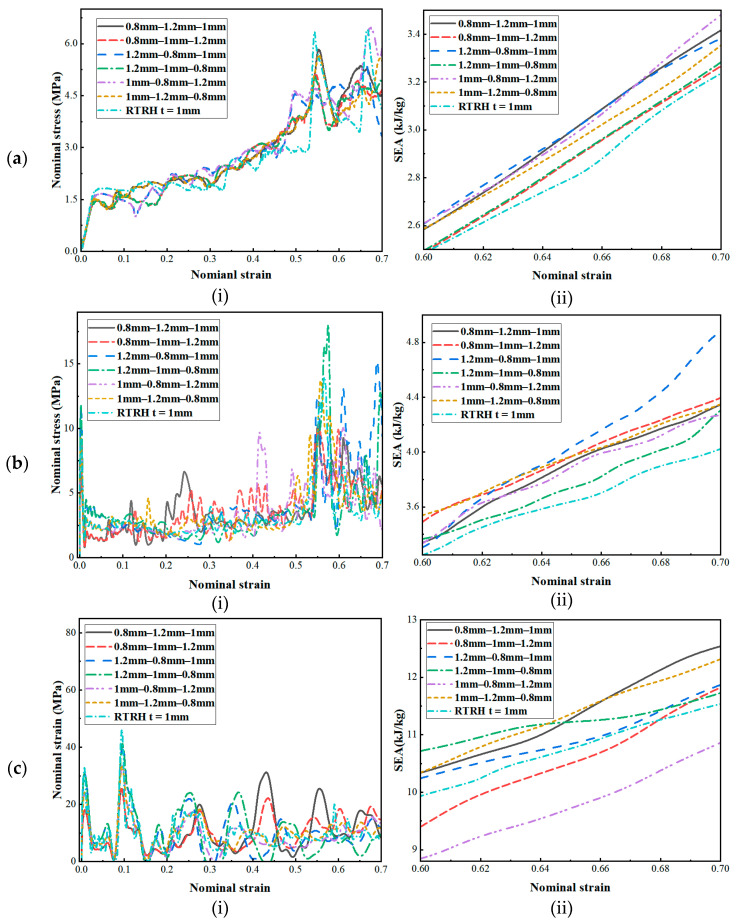
Dynamic response of the RTRH for different wall thickness gradients. (**a**) *v* = 1 m/s, (**b**) *v* = 30 m/s, and (**c**) *v* = 100 m/s.

**Figure 22:**
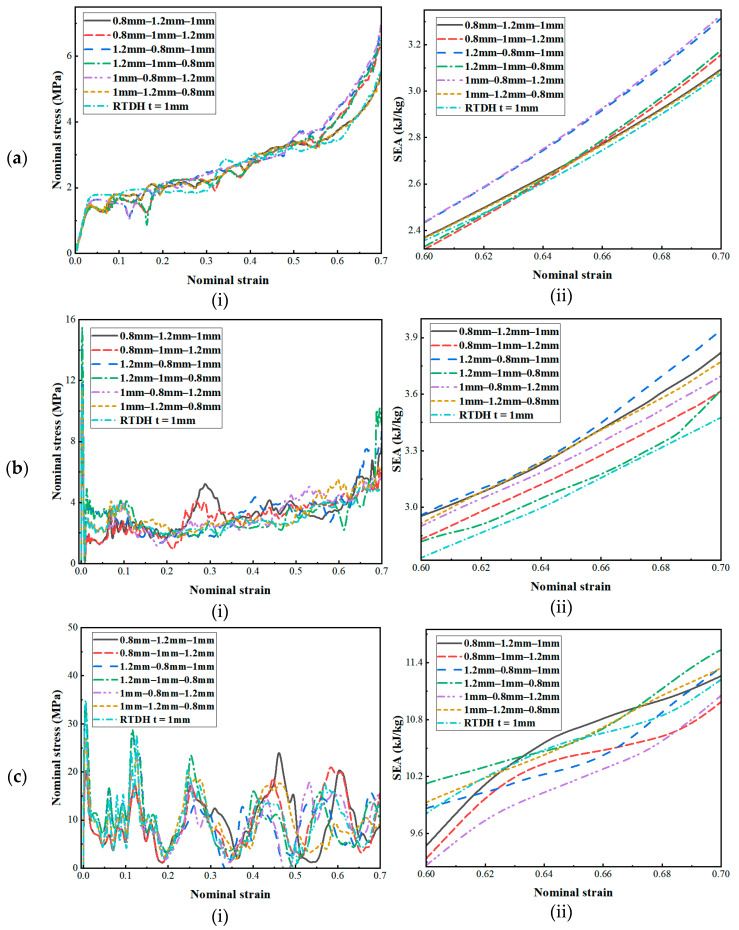
Dynamic response of the RTDH for different wall thickness gradients. (**a**) *v* = 1 m/s, (**b**) *v* = 30 m/s, and (**c**) *v* = 100 m/s.

**Figure 23:**
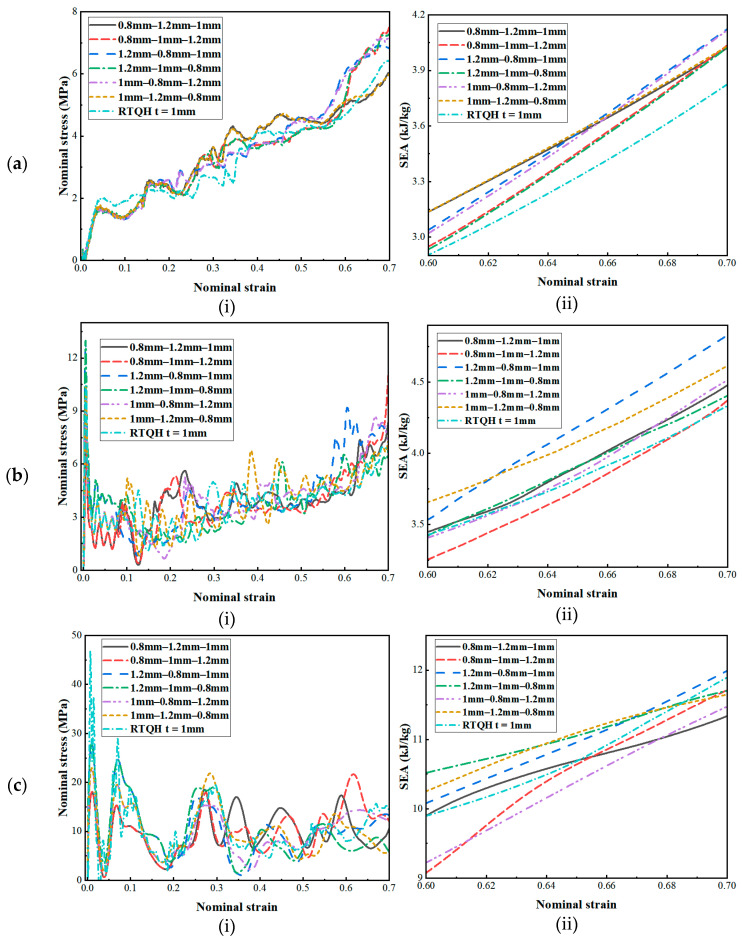
Dynamic response of the RTQH for different wall thickness gradients. (**a**) *v* = 1 m/s, (**b**) *v* = 30 m/s, and (**c**) *v* = 100 m/s.

The geometrical parameter *t* is a quantity related to the wall thickness of the honeycomb structure. The wall thickness of the honeycomb structure is closely related to its energy absorption characteristics. When the wall thickness of the structure increases, the energy absorption capacity of the honeycomb structure first increases and then decreases [5]. Therefore, in this section, the thickness of the honeycomb structure was optimised in conjunction with the gradient theory, and six different thickness gradients were designed without changing the weight of the honeycomb structure, as shown in Figure 20. The dynamic responses of the three structures under different gradients obtained by finite element simulation are shown in Figure 21, Figure 22 and Figure 23.

As can be seen from Figure 21a, Figure 22a and Figure 23a, when the velocity was low, the structural stress difference under different wall thickness gradients was not very large, and the specific absorption energy was slightly larger than that of the uniform wall thickness structure. However, as the velocity reached the medium-velocity impact, as shown in Figure 21b, Figure 22b and Figure 23b, the structural stress difference of different gradients gradually appeared, and the specific absorption energy also produced a large change, in which the negative mixed-thickness gradient (1.2 mm–0.8 mm–1 mm) (remembering that the wall thickness from large to small is a negative gradient, and that from small to large it is a positive gradient) in the medium-velocity impacts of the three structures exhibited a more excellent specific energy absorption performance than that of the uniform-thickness structure’s energy absorption performance. As shown in Figure 21c, Figure 22c and Figure 23c, the difference in energy absorption between different wall thickness gradients gradually increased under high-velocity impacts, from which it can be seen that, compared with the uniform-thickness RTRH, RTDH, and RTQH structures, the positive-mixed-thickness gradient (0.8 mm–1.2 mm–1 mm), the negative-thickness gradient (1.2 mm–1 mm–0.8 mm), and the negative-mixed-thickness gradient (1.2 mm–0.8 mm–1 mm) showed good energy absorption capacity, respectively.

## 4. Conclusions

Aiming to address the problem of poor energy absorption due to the limited compression deformation in the rotating, rigid body structure within the negative Poisson’s ratio structure, this paper designed the NRTS structure by integrating the concave structure into the rotating rigid body cell and systematically investigated the dynamic response of the NRTS structure through various theoretical analyses, physical experiments, and numerical simulations. Due to the differences in the rotation angles of the NRTS structure, the effects of different rotation angles are discussed in this paper. Finally, based on the gradient theory, a hybrid cell gradient design and a wall thickness gradient design were applied to the NRTS structure and compared using finite element simulation. Through the above studies, the following conclusions were obtained in this paper:

- In this paper, finite element models of three NRTS structures were designed. Then, samples of RTRH were fabricated through 3D printing. In this study, the stress-strain curves of RTRH in quasi-static compression experiment and finite element simulation were compared. The error between the experimental and finite element simulation results was found to be 6.6%. This proves that the finite element model developed in this paper can effectively simulate real working conditions. These findings provide a reference for the design of honeycomb structures.
- Compared to the RH and RTST structures, the NRTS structure was shown to have a good energy absorption capacity. In low-velocity impact, the RTQH and RTRH structures demonstrate higher plateau stresses. Compared to the RH structure, the RTQH and RTRH increased by 78% and 124%, respectively. When compared to the RTST structure, the increases are 20% and 51%, respectively. Compared to the RH and RTST structures, the RTQH structure shows an increase in energy absorption by 21% and 20%, respectively. The RTDH structure exhibits an excellent CEF, which is 54% and 57% higher compared to the RH and RTST structures, respectively. The RTQH and RTRH structures have the highest plateau stresses at medium-speed and high-speed impacts, respectively.
- For the RTRH structure, different rotation angles have a significant effect on the deformation pattern and energy absorption capacity. The RTRH structure has excellent energy absorption at a rotation angle of 20° for low- and medium-speed impacts, and even better energy absorption at a rotation angle of 40° for high-speed impacts. In the case of the RTDH and RTQH structures, the rotation angle does not affect the deformation pattern. The effect of different rotation angles on the energy absorption performance is less significant at low- and medium-impact speeds, whereas for high speed impacts, the RTDH-40°and RTQH-30° have better energy absorption.
- At low-impact velocities, the gradient structures are not significantly different from each other, but the specific absorption energies are all slightly higher than those of the uniform gradient structures. As the impact speed increases, the differences between the gradients gradually appear and expand. Among them, the B-A-C structure, the positive mixed thickness gradient (0.8 mm–1.2 mm–1 mm), the negative thickness gradient (1.2 mm–1 mm–0.8 mm), and the negative mixed thickness gradient (1.2 mm–0.8 mm–1 mm) all further increase the energy absorption capacity of the NTRS structure.

## Figures and Tables

**Table 1:** Relative density of honeycomb structures.

Honeycomb Structure Name	$\bar{\rho }$
RTRH	$\frac{4(l_{1}\sin\theta_{1}+h_{1})t}{[2l_{1}h_{1}+(l_{1}{}^{2}+h_{1}{}^{2})\sin2\theta_{2}]\sin\theta_{1}}$
RTDH	$\frac{2(2l_{1}\sin\theta_{1}+\pi h_{1})t}{[2l_{1}h_{1}+(l_{1}{}^{2}+h_{1}{}^{2})\sin2\theta_{2}]\sin\theta_{1}}$
RTQH	$\frac{\pi (l_{1}{}^{{}^{2}}+h_{1}{}^{2})t}{2[2l_{1}h_{1}+(l_{1}{}^{2}+h_{1}{}^{2})\sin2\theta_{2}]\sin\theta_{1}}$
RH	$\frac{2(l_{2}\cos\theta_{3}+h_{2})t}{(2l_{2}\cos\theta_{3}-h_{2}\sin\theta_{3})h_{2}}$
RTST	$\frac{4(l_{1}+h_{1})t}{2l_{1}h_{1}+(l_{1}{}^{2}+h_{1}{}^{2})\sin2\theta_{2}}$

Where *t* is the wall thickness of the honeycomb structure.

**Table 2:** Structural parameters of each honeycomb.

Honeycomb Structure Name	l (mm)	h (mm)	a (mm)	θ1 (°)	m (kg)	θ2 (°)	t (mm)	d (mm)
RTRH	14.61	14.61	4.23	60	0.124	30	1	20
RTDH	14.61	14.61	4.23		0.127	30	1	20
RTQH			3.03		0.128	30	1	20
RH	15.77	20		60	0.170		1	20
RTST	14.61	14.61			0.115	30	1	20

**Table 3:** Honeycomb structure platform stresses.

Honeycomb Structure Name	$\varepsilon_{y1}$	$\varepsilon_{d1}$	$\varepsilon_{y2}$	$\varepsilon_{d2}$	$\sigma_{p}$
RTRH	0.03	0.33	0.356	0.72	5.52
RTDH	0.04	0.3	0.344	0.6	4.94
RTQH	0.045	0.34	0.3575	0.72	6.95
RH	0.018	0.68			3.1
RTST	0.035	0.31	0.33	0.78	4.6

**Table 4:** SEA of the RTDH structure with different rotation angles (kJ/kg).

	Structure	RTDH-20°	RTDH-30°	RTDH-40°
Velocity				
1 m/s		2.95	3.08	2.97
30 m/s		3.49	3.48	3.37
100 m/s		10.62	11.22	11.56

**Table 5:** SEA of the RTQH structure with different rotation angles (kJ/kg).

	Structure	RTQH-20°	RTQH-30°	RTQH-40°
Velocity				
1 m/s		3.69	3.82	3.69
30 m/s		4.22	4.33	4.21
100 m/s		10.96	11.88	11.10

## Data Availability

The author has uploaded the main data to the system. If in doubt, the author can be contacted by email (13832240299@163.com, Nuo Chen).
